# A More Efficient Transportable and Scalable System for Real-Time Activities and Exercises Recognition

**DOI:** 10.3390/s18010268

**Published:** 2018-01-18

**Authors:** Kévin Chapron, Valère Plantevin, Florentin Thullier, Kévin Bouchard, Elise Duchesne, Sébastien Gaboury

**Affiliations:** LIARA Laboratory, Université du Québec à Chicoutimi, Chicoutimi, QC G7H 2B1, Canada; valere.plantevin1@uqac.ca (V.P.); florentin.thullier1@uqac.ca (F.T.); kevin.bouchard@uqac.ca (K.B.); elise1_duchesne@uqac.ca (E.D.)

**Keywords:** activity recognition, wearable, power efficiency, machine learning

## Abstract

Many people in the world are affected by muscle wasting, especially the population hits by myotonic dystrophy type 1 (DM1). Those people are usually given a program of multiple physical exercises to do. While DM1 and many other people have difficulties attending commercial centers to realize their program, a solution is to develop such a program completable at home. To this end, we developed a portable system that patients could bring home. This prototype is an improved version of the previous one using Wi-Fi, as this new prototype runs on BLE technology. This new prototype conceptualized induces great results.

## 1. Introduction

Myotonic dystrophy type 1 (DM1) is among the most frequent neuromuscular disorders worldwide. The highest prevalence of that neuromuscular disorder is recorded in the Canadian province of Québec. The sheer size of the affected population creates a perfect environment to study its complex properties [1]. People with DM1 display an important number of heterogeneous symptoms. For instance, they can develop cataracts, insulin resistance, or even cardiac conduction disorder. In addition, skeletal muscles, the category of muscles which are generally attached to the bones by arrays of collagen fibers, are largely afflicted. This aspect of the disease has been demonstrated to be declining by one to three percent per year in recent studies [2]. The atrophy of the lower-extremity muscles can result in a significant impact on the life quality of patients. Moreover, it has been reported as a strong predictor of disrupted social participation [3]. Although the muscle-wasting is largely present in this population, it can be observed in many others populations, such as elderly people linked to muscle strength declining over the years [4]. Since no curative therapy currently exists for these patients, researches focus on developing interventions that could attenuate the muscle wasting. For example, strength-training, which is a type of physical exercises specializing the induction of muscular contraction through the use of resistances, has been shown to be a safe and efficient way to increase muscles strength. However, the apathy reported in the DM1 population (40%) [5] represents a major factor that could jeopardize their participation to such a highly demanding strength-training program.

In order to address this situation, researchers focus on the development of a strength-training program which could be followed at home by the patient. While the idea seems simple, many barriers limit such development. To be useful, the program would need to be closely monitored for quality and efficiency assessment by health professionals. Indeed, when a physical therapist establishes a physical program for a patient, he wants to know whether the person follows it in a rigorous and consistent manner or not. Obviously, performing the strength-training program in the patient’s home causes challenges for the therapist to supervise it. The human resources required for this task is prohibitive, especially in a context of healthcare economics scarcity.

A potential solution to enable at-home training lies in the exploitation of ambient technologies through the development of a system using machine learning and wearable sensing technologies [6]. To do so, proposed hardware solutions have to be as small as possible since patients must be able to take it to their home easily. Hence, wearable apparatus seems like a viable solution with a high potential to address the task at hand. In the literature, researchers have already suggested various wearable technologies, such as smart watches, to recognize postures [7] or gestures [8] in real-time. The real potential of this type of technology could be achieved by implementing either novel techniques or algorithms to provide assistance and guidance to the patient while recognizing his physical activities. Guidance could take several forms such as advices on how to perform an exercise, or even vocal encouragement messages to support the person during the program. Such system would need to take into account the major motivational stakes lived by this population to be effective.

The main task of this new prototype is to recognize the ongoing activities performed by the patient. This is a classical instance of the well-known activity recognition problem [9]. This problem can be defined as the mapping of extracted data from a set of sensors to an activity model for a certain period of time [10]. In this particular instance of the problem, the goal is to recognize in real-time each individual physical exercises which are part of a program built by a physical therapist. The health professionals also desire to be aware of the activity level, in quantitative terms, of the patient during a day, or during other specified time-intervals. Activities that requires to be monitored, in that case, would be broadly defined, including, for example, walking, running, sitting, or standing. Furthermore, since daily activities are performed on short periods of time (i.e., “standing” is a short action) and physical exercises run on lengthier periods of time, it induces a challenging issue: recognizing activities on two different time frames with the same device. Despite the amazing progress show in recent years [11], commercially available devices are not able to perform such a difficult task. However, the hardware necessary for this task exists, and therefore, customized wearable devices [12] could be used to recognize all these activities.

In our previous work [13], we designed a system coupling a *Raspberry Pi 3* and a computer-on-module *Intel^^®^^ Edison* which was able to perform real-time activity recognition of some basic daily activities (walking, running, sitting, standing) but also some specific exercises (front step down, squat on chair, front lunge, sit to stand) with an average recognition rate of 99% using a Random Forest classification algorithm. The *Intel^^®^^ Edison* was provided with a 9 axis Inertial Measurement Unit (IMU) module and was attached to the wrist to collect and stream raw inertial data (at a stable frequency of 100 Hz) to the *Raspberry Pi 3* which was responsible to process the received data, compute features, perform activity recognition and broadcast the result of this recognition through WebSocket. The communication between the *Intel^^®^^ Edison* and the *Raspberry Pi 3* was made over Wi-Fi. Although the proposed system worked very well, a major issue refrained it from being used in real case scenarios. The Wi-Fi communication between the two devices is overly energy consuming. In fact, the battery life of the *Intel^^®^^ Edison* was approximately of two and a half hours which is clearly insufficient for recognizing activities executed during a full day. Therefore, in order to have a viable solution as regards battery life, we decided to design and build a very low-cost wrist-worn device provided with a 9-axis IMU communicating with the *Raspberry Pi 3* through Bluetooth Low Energy (BLE) and having a battery life of nearly a day in worst cases. In this paper, we present our new real-time activity recognition system consisting in this new wrist-worn device and a *Raspberry Pi 3* that has been thoroughly compared with the previous one [13] by conducting the same experiment over the same participants.

The rest of the paper is structured as follows: Section 2 reviews the literature about activity recognition using inertial data. Moreover, this section also provides an overview of proposed commercial solutions. The third section recalls the previous system we suggested by describing its structure. Next, Section 4 provides a detailed description of the design, regarding both hardware and software, of the new version of the wearable device we suggest in this paper. In Section 5, we present the activity recognition algorithm and the different features required. Section 6 describes the experiments that have been conducted to determine the efficiency of our proposed method. The results obtained are showed in Section 7 and discussed in Section 8. Finally, Section 9 concludes the paper by synthetizing the contribution and by presenting the future avenues of research.

## 2. Related Work

Human activity recognition is a very active area of research. With the arrival of several types of wearable devices equipped with different kinds of sensors (e.g., accelerometer, gyroscope, temperature sensor, altimeter, and optical sensor), researchers have put a lot of effort into developing algorithms and techniques to recognize physical activities performed by persons. Combining wearable devices and data mining techniques to recognize activities has proven to be very effective in the recent years [14]. In order to position our work, it was decided to divide the related works into two parts. First, the works that do not involve smartphones but only accelerometers worn directly on the person are reviewed. Second, the commercial wristbands enabling different level of activity recognition are discussed.

There are plenty of researchers working with smartphones containing an accelerometer to collect data [15,16]. This is a relatively straightforward way to collect data considering that most of the population have a smartphone nowadays. However, this assumption does not hold true with the population suffering from DM1. Indeed, this population is often financially challenged and is, therefore, limited in terms of buying power. Accordingly, it seems compelling to review the work on physical activity recognition that do not involve expensive devices such as smartphones but solely accelerometers worn directly on the person. To begin with these researches, Khan et al. [6] used a single chest worn accelerometer to recognize daily activities (standing, sitting, walking, running, etc.). Their device streams the raw data from the accelerometer to a computer using Bluetooth at a 20 Hz frequency. They obtained an overall accuracy of 97.9% using an Artificial Neural Network (ANN). However, their method only focused on daily activity recognition. In their work, Mannini et al. [17] compared the recognition accuracy obtained from two placements (wrist, ankle) of a single accelerometer. They compared these two positions by testing them with many exercises such as cycling, sitting, painting, lying. Obviously, they observed better results when the device was placed on the most active part of the body depending on the exercise (e.g., ankle for cycling, wrist for painting). As a result, they obtained an overall accuracy of 84.7% with the wrist, and 95.0% with the ankle. Next, the study from Cleland et al. [18] investigates the many other possible placements for a single inertial measurement unit (IMU). They compared the placement of the IMU on the chest, the lower back, the hip, the wrist, the thigh and the foot, with a sampling rate of 50 Hz to recognize daily activities. They also studied recognition performance of several popular classifiers: Support Vector Machine (SVM), Decision Tree (DT), Naïve Bayes (NB) and ANN. They found when the IMU was placed at the wrist that the best recognition rate (95.88%) was reached when using SVM as classifier. Unfortunately, they did not assess the recognition performance of two frequently encountered algorithms in activity recognition which are k-nearest neighbors (k-NN) and Random Forests (RF). Using RF as classifier, Ellis et al. [19] showed a recognition rate of 94% for the following daily activities: sitting, standing, walking, bicycling and being in a vehicle. They utilized a device composed of an accelerometer and a GPS worn on the hip and the experiments was conducted in a highly controlled environment. One last interesting work is that of Cheng et al. [20]. They used multiple accelerometers to capture data when a person was performing daily activities and few physical exercises (e.g., dumbbell, bench dips, and squats). Their system achieved a recognition accuracy of 79% using their custom algorithm and sampling the data at a 100 Hz rate.

For the second part of this literature review, we turn our focus toward our contextual needs. Previously, our team worked to develop a working prototype involving a wristband. This first prototype worked as a proof-of-concept; it demonstrated the feasibility of recognizing both activities and physical exercises on the same device despite their inherent differences in temporal granularity. Other commercial and academic apparatus exist in the literature. It seems important to discuss about them and compare them with the first prototype that was developed in our laboratory. Among the plenty of wristbands which integrate inertial sensors, a very popular one is the FitBit [21]. The battery of this device can retain charge for about 7 continuous days. It can recognize daily activities such as walking, running, biking, and can also two distinct categories of physical exercises: sports and aerobic workout. Yet, it does not recognize exactly which physical exercise the wearer does. Moreover, while this wristband can distinguish many activities, the raw data from its sensors cannot be extracted which limits the potential of the device for research. Lastly, it is relatively expensive with a price sitting around 180 USD. Another trendy wristband is the Garmin vívosport™ [22]. Its battery can hold up to 7 days while recognizing physical activities like swimming, running, biking. Similarly to Fitbit, the data from this device are completely invisible for the user, and cannot be extracted neither. In addition, the price of this wristband is even higher than the FitBit towering around 200 USD. The next wristband identified is the Polar Loop [23]. It can recognize the number of steps walked and an hour of race training for each day. The data cannot be extracted from this device either. Still, the wristband can operate up to 8 consecutive days considering around one hour of training a day. The recognition module is more limited than the vívosport™ and the FitBit, but its price is also much lower. It can be purchased for around 95 USD. Last but not least, with the E4 wristband [24], third party user can collect the raw data easily through the Bluetooth protocol. Besides, its internal memory can allow a storage of 36 h of data. Its battery can hold 20 h while streaming its data, or 36 h or more while recording the data on its internal storage. Despite this major advantage, the device itself does not recognize anything. Moreover, the wristband is the most expensive available on the market with a prohibitive price of more than 1600 USD.

In summary, a large portion of the literature is based on accelerometers worn at the wrist. These approaches all show impressive recognition accuracy for daily activities such as walking, running, standing, and sitting. However, very little work has been done on recognizing specific physical exercises. In fact, except the work from Cheng et al. [20] there are no other approaches, to the best of the authors knowledge, that recognize both daily activities and specific physical exercises on the same device. The major drawback of the method of Cheng et al. [20] is that it requires the use of multiple accelerometers worn at different locations on the person. There are two other critical points to consider in our context. On one hand, the cost of the device must be as low as possible in order to be affordable by the DM1 population. On the other hand, the device employed must allow accessibility to the raw data in real-time. As mentioned above, almost all commercial devices do not allow access to the raw data and are very expensive. The only one corresponding to our needs would have been the E4 wristband which is definitely too expensive for the targeted population. In this paper, we present an affordable system composed of a low-cost wrist-worn device provided with a 9 DOF IMU and a Raspberry Pi 3 communicating via Bluetooth Low Energy that recognizes very efficiently daily activities (walking, running, sitting, standing) as well as four exercises (front step down, squat on chair, front lunge, sit to stand).

## 3. Previous System

In our previous work [13], we presented a Proof of Concept (PoC) using two different devices. The first one was a wearable device based on the *Intel^^®^^ Edison* IoT platform. It was employed to collect the data stream produced by an Inertial Measurement Unit (IMU)—a *LSM9DS0*. These data were then sent, over Wi-Fi, from the wearable device to a *Raspberry Pi 3*, which was considered as the main computing unit. In that sense, this section recall, in brief details, the design of our previous PoC.

The *Intel^®^ Edison* is an Internet of Things (IoT) platform that embeds an *Intel^®^ Atom™* dual-core processor clocked at 500 MHz as well as 4 GB of flash storage. The distributor *Sparkfun* provides several supplementary blocks such as a SD card block or a 9-axis IMU block. Such additional pieces of hardware allow to improve the base functionalities as well as the ease of use of the *Edison* since each block may be stacked to build a more complex and powerful device. Through this, it was possible for us to make the *Intel^®^ Edison* platform fit our needs since at least a 9-axis IMU and a power supply were required to build the prototype. However, few additional blocks such as a micro SD card slot were also desired. The Table 1 provides details of each part of the previously proposed wearable device prototype that was built upon the *Intel^®^ Edison* platform.

**Figure 1:**
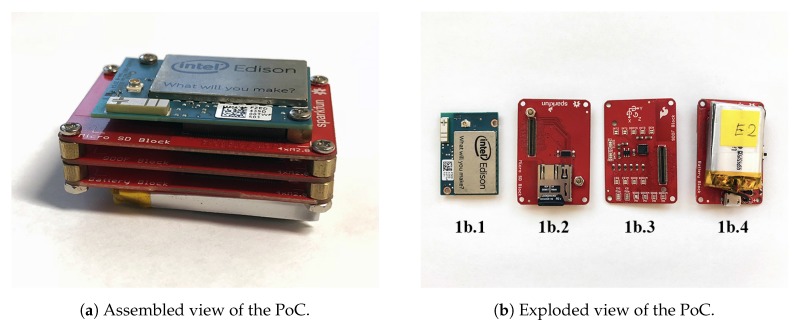
Previously proposed wearable device for real-time activity and exercises recognition. From left to right the *Intel^®^ Edison* (1b.1), the micro SD card (1b.2), the 9-axis IMU (1b.3) and the power supply blocks (1b.4).

The Figure 1 shows both the assembled view (Figure 1a) of our early prototype as well as an exploded view (Figure 1b) to showcase every individual component that were detailed in Table 1. To make such a device become a wearable one, a *Velcro* strap was hooked properly all around the assembly in order to secure its position on the wrist. The final dimension for the wearable device was: 45 mm (height) × 32 mm (length) × 22 mm (depth).

Since we desired to recognize activities and exercises through machine learning algorithms by exploiting inertial data, the complexity of such methods is overly expensive as regards required computation resources. Consequently, processing them directly on the *Intel^®^ Edison* platform with the actual development of the code is not possible since it would require deep optimization. In that sense, the computation of the recognition needed to be handled by a more powerful computing unit. However since the main objective of our work was to help people performing their physical program at home, such an entity had to be as inexpensive as possible, portable and simple to integrate in an existing environment. Therefore, we opted for a *Raspberry Pi 3* to fulfill this task as it met every of our needs. Moreover, since such an embedded computer is already used in several domains such as IoT or Education [25] it is possible for us to guarantee its reliability.

Through our previous research [13] we exposed that this experimental system was accurate and viable to perform a real-time recognition of both activities and exercises. Nevertheless, we observed that the significant amount of power consuming for the wearable devices was a major drawback. Such an issue was mainly due to a native high power consumption of the *Intel^®^ Edison* platform as well as the use of the Wi-Fi technology to stream data from the 9-axis IMU (≈80 mA and ≈160 mA when the Wi-Fi is both disable and enable, respectively). Hence, we observed that the battery was able to power the wearable device for only two hours and a half, that is clearly not satisfying for a daily usage in real use-case scenarios.

## 4. A Most Efficient System

As recalled in the previous section, our previous system experienced a major drawback especially regarding the power-consumption of the wearable device. Consequently, we expressed the necessity to improve its design to overcome such an energy efficiency issue. To this end, this section describes in details the changes brought to the novel system and particularly to the wearable device both in terms of hardware and software in order to suggest a new power-efficient wearable device prototype.

### 4.1. Hardware

**Figure 2:**
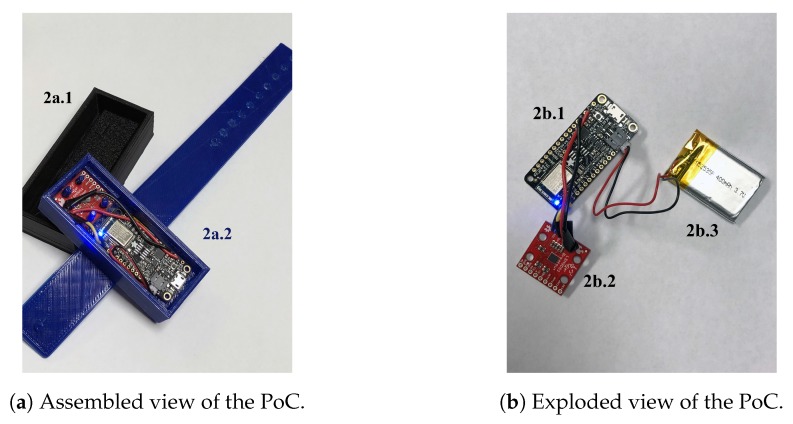
Novel wearable device designed for real-time activity and exercises recognition. From left to right the PLA cover (2a.1), the flexible wristband including the electronic casing (2a.2), the *Adafruit Feather nRF52 Bluefruit LE* development board (2b.1), the *LSM9DS1* 9-axis IMU (2b.2) and the 400 mAh power supply (2b.3).

The new wearable device is based on the *nRF52* microcontroller (i.e., *nRF52832*). This chip was chosen in accordance with the specification provided by its manufacturer (i.e., *Nordic Semi Electronics*) since it is “ideally suited for ultra low-power wireless applications”. Indeed, the *nRF52* embeds the Bluetooth^®^ Low Energy (BLE) technology that aims at replacing the Wi-Fi technology employed in our previous prototype in order to stream inertial data. As a consequence, the power efficiency should be better preserved with such a change. Moreover, since this microcontroller offer both an SPI as well as an I^2^C bus, it let us employ every required modules as the previous device (i.e., mainly a 9-axis IMU only in this new prototype since the SD card slot was no longer desired). Figure 2 depicts both the assembled view (Figure 2a) of our new prototype and an exploded view (Figure 2b) to exhibit every individual component.

### 4.2. Software

In order to perform the data collection, a piece of software had to be designed first. In that sense, the embedded firmware we developed is capable of reading the values from the IMU that is wired through a standard I^2^C lane to the *nRF52* and stream them to a *Raspberry Pi 3* using the BLE protocol at a precise sampling rate of 60 Hz.

Firstly, each sensor of the IMU require an initial setup to scale their ranges. Therefore, the three main parameters were defined as follows: ±4 g for the accelerometer; ±2000 °/s for the gyroscope and ±4 gauss for the magnetometer. Next, since we expressed the need to drastically reduce the power consumption of our device, we moved to the Bluetooth Low Energy (BLE) technology to cope with the huge amount of power needed by the previously used Wi-Fi protocol. This choice was mainly motivated because such a technology was meant to enable low power wireless communications [26]. This protocol works as a client/server architecture where each data is stored in a characteristic and flagged as *readable*, *writable* or *notifiable* depending if the client have to read the data, write in it or subscribe to its changes. The last one is the most interesting as it allows to stream data. The main problem with this kind of streaming is the sampling rate. Hence, to preserve the best possible frequency, the fewest number of characteristics has to be exploited. In that sense, we decide to deal with only a single one to stream the whole set of data. Although it is possible for a characteristic to notify values of 20 bytes long, the IMU of our wristband returns 9 floats encoded on 4 bytes, resulting in 36 bytes of data in total. Thus, it appeared essential to develop a compression mechanism to make inertial data produced by the IMU fits in a single BLE characteristic.

To suggest an optimal data compression, we first define the number of significant digits that are possible to be provided by the IMU. Since the *LSM9DS1* holds sensitivities of $10^{-3}$ g for the accelerometer, $7\;\times \;10^{-2}$ °/s for the gyroscope and $14\;\times \;10^{-2}$ gauss for the magnetometer, both the accelerometer and the magnetometer are capable of providing 4 precision digits values while the gyroscope offers a float value of only one precision digit. Hence, it seems that a full float precision is not required to store these inertial values. Moreover, through some tests we have conducted, a 3 precision digits for both the accelerometer and the magnetometer was determined as valuable. In a nutshell, we keep 6 values with 3 precision digits (*x*, *y* and *z* axis for both the accelerometer and the magnetometer), as well as 3 values with only one digit (*x*, *y* and *z* axis for the gyroscope). Then, all of these values are in open intervals defined by the IMU configuration that are: $]-4,4[$ for the accelerometer and magnetometer and $]-2000,2000[$ for the gyroscope. From this point, these float values were transformed to integer by using both a decimal shifting method and a truncation of the float part that is outside the required precision. Through such a conversion, the inertial data are now included in the following intervals: $]-4000,4000[$ for the accelerometer and magnetometer and $]-20000,20000[$ for the gyroscope.

**Figure 3:**
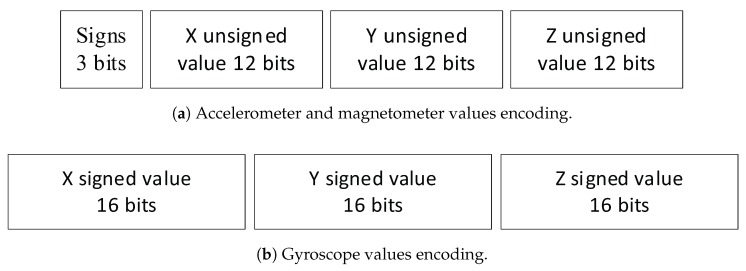
Graphical representation of a packet containing the whole set of compressed data produced by the IMU that is transmitted over a single BLE characteristic to the main computing unit.

Finally, the data compression that was applied on the values produced by every sensors of the 9-axis IMU let us consume only 13 bits (i.e., 12 for the value and 1 for the sign) to encode both the values of the accelerometer as well as the magnetometer and 16 bits are needed for the gyroscope (i.e., 1 bit for the sign and 15 for the value). Accordingly, as exposed in Figure 3, the resulting packet have a total size of 87 bits being 11 bytes that refers to the half of the maximum size for values of one BLE characteristics. In that sense, the firmware of our novel prototype we suggest in this paper is capable of streaming the whole set of data produced by the *LSM9DS1* through a single characteristic.

## 5. Recognition

Once compressed data are received by the *Raspberry Pi 3*, the first task that have to be completed by such a main computing unit is to reverse the compression process in order to obtain the decompressed raw data. Then, depending on the recognition that have to be achieved, a feature extraction is performed over both daily activities or specific exercises raw signals before classify them with a machine learning algorithm. This section describes the whole set of features that are computed as well as the selected machine learning algorithm employed to achieve the classification process.

### 5.1. Feature Extraction

The features extraction process must be achieved before classify them with a machine learning algorithm. In that sense, we opted for 70 well-known features [8,15,16,18,27,28,29,30], that came from both time and frequency domains. Depending on the type of action the system has to recognize (i.e., daily activities or specific exercises), the features are computed from a fixed time window of either 2 or 6 s with 50 % overlapping over each raw signal since it has been proven that such a configuration performs well [8,17,18,31].

On the first hand, the time-related ones are composed of 11 statistical computations. The first operation is a simple unweighted mean on each axis for each sensor (i.e., gyroscope, accelerometer and magnetometer). Then, the average of these values for each specific sensor is processed. In the exact same way, we apply few more statistical calculations: the standard deviation, the *skewness*, the *kurtosis*, the *Zero-crossing rate* and finally, the correlation between all possible axis combinations. Formulas of the skewness and the kutosis measures, when applied to the *W* axis are respectively reminded in (1) and (2). In both cases, *N* represents the number of elements in the vector, $w_{i}$ is the *i*th element in it, $\bar{W}$ is the mean of the vector and $\sigma_{w}$ refers to its standard deviation. Finally, we calculate the correlation between each axis combination from the same sensor as recalled by (3).

(1) $$skew\left(W\right)=\frac{N}{\left(N-1)(N-2\right)}\;\cdot \;\left(\sum_{i=1}^{N}\frac{{\left(w_{i}-\bar{W}\right)}^{3}}{\sigma_{w}^{3}}\right)$$

(2) $$kurt\left(W\right)=\frac{N(N+1)}{\left(N-1)(N-2)(N-3\right)}\;\cdot \;\left(\sum_{i=1}^{N}\frac{{\left(w_{i}-\bar{W}\right)}^{4}}{\sigma_{w}^{4}}-\frac{3{\left(N-1\right)}^{2}}{\left(N-2)(N-3\right)}\right)$$

(3) $$\rho \left(U,V\right)=\frac{cov(U,V)}{\sigma_{u}\;\cdot \;\sigma_{v}}$$

On the second hand, several attributes required to be transformed from the time domain to the frequency one. In order to achieve such a conversion, the Fast Fourier Transform (FFT) algorithm has been used. These three features are the DC component, the spectral entropy for each acceleration axis and finally, the Energy that is defined in (4), where *W* represent a vector of complex numbers, *N* refers to its size and $w_{i}$ is the *i*th element in it.

(4) $$energy\left(W\right)=\frac{1}{N}\;\cdot \;\left(\sum_{i=1}^{N}\left|w_{i}\right|\right)$$

### 5.2. Classification

Once discriminating features extracted from raw inertial data are computed, the following task that must be achieved by our system is a classification process. According to the literature in the field of activity recognition, it is known that several of such algorithms achieve excellent results in classifying data from inertial sensors. As examples, we can mention: Decision Trees (DT), *k*-Nearest Neighbors (*k*-NN), Support Vector Machines (SVM), Artificial Neural Networks (ANN), and Naïve Bayes [18].

In that sense, this research suggests to conserve the same classifier that was employed in our previous research, the Random Forest algorithm. As a non-parametric method (method that do not make strong assumptions about the form of the mapping function), this algorithm remains simple, flexible, powerful and efficient [32]. Indeed, its satisfying classification performance was reported in the related literature [18,19] as well as by our previous work [13], where the same set of features extracted from inertial data was the input of this classifier.

**Figure 4:**
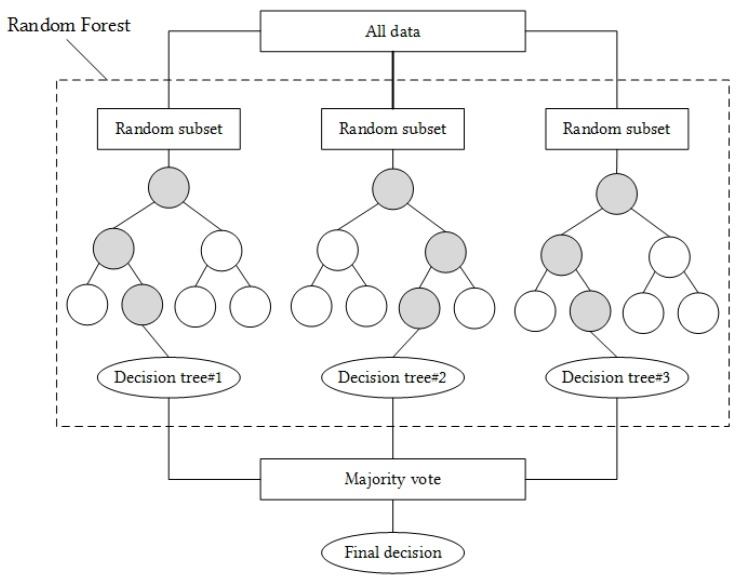
A simple example of a Random Forest classification using B=3 trees.

According to Breiman [33], a Random Forest (RF) is a combination of tree predictors, where each tree depends on the values of a random vector, sampled independently and with the same distribution for all trees in the forest. The final decision is made by a majority vote between each outcome of each single tree that forms the forest. Figure 4 exposes a simple example of a Random Forest classification using three trees. Such a classification algorithm implies several initial parameters. The three most important ones which are necessary to define are first, the number of trees in the forest (*B*), second, the number of features to consider when looking for the best split (*F*) and the function used to measure the quality of such a split (*C*). According to the literature, Breiman [33] suggests using $F=log_{2}\left(m\right)+1$ where *m* refers to the number of attributes present in the dataset. However, it is also possible to find other recommendations such as $F=\frac{1}{2}\sqrt{m}$ or $F=\sqrt{m}$. As regards the quality of the split, the two primary function often employed are first, the calculation of the Gini index and second, the evaluation of the information gain that are respectively expressed by (5) and (6):

(5) $$Gini\left(T\right)={1-\sum_{i=1}^{n}\left(p_{i}\right)}^{2}$$

(6) $$E\left(T\right)=\sum_{i=1}^{n}-\left(p_{i}log_{2}p_{i}\right)$$

where *T* are input data containing instances of *n* classes and $p_{i}$ is the relative frequency of class i∈n in *T*. However, this criterion depends essentially of the type of trees that are used in the construction of the forest (i.e., ID3, C4.5, CART, etc.).

## 6. Experiments

First of all, the experiments we have conducted involved several participants which are described in the first point of this section. The following point describes in details the procedure of the data collection that each participant were asked to perform.

### 6.1. Participants

Since our previous work, only 10 over the 13 recruited participants were available. They were all the same university students and professor, being 1 female and 9 males, aged from 23 to 30 years old [13]. All of them were healthy people without any motor function issues.

### 6.2. Exercises and Daily Activities

**Figure 5:**
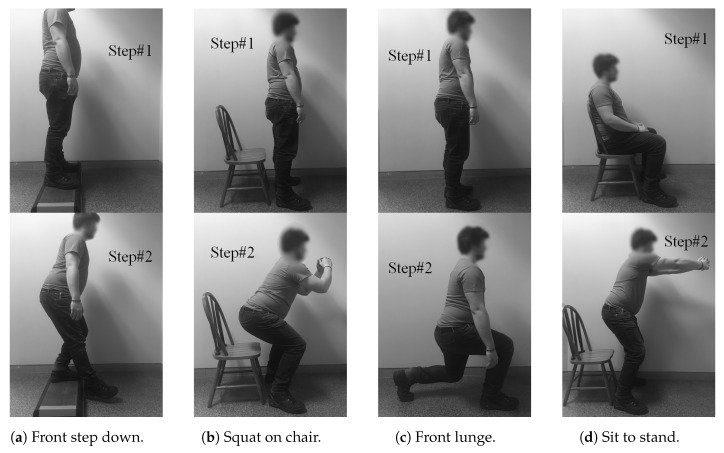
Full set of specific exercises selected from a real program prescribed by therapists to their patients suffering from neuromuscular problems.

As we aim at designing a wristband capable of recognizing both daily activities as well as specific exercises, it was essential to define the most valuable ones to be performed by our participants in order to validate the accuracy of our system. In that sense, we selected a real program that therapists already prescribe for their patients suffering from neuromuscular problems. Figure 5 shows the four exercises that form such a rehabilitation program. For each practice, the movement (i.e., the second step) have to be hold for two seconds at most and finally, as soon as the training session is completed, the patient is returning back to his/her daily activities. As concerns daily activities, we only focus on the five predominant ones including *walking*, *running*, *sitting down*, *standing up*, *stay seated*.

### 6.3. Procedure

To perform the data collection, the first step involved to ask each participant to position himself or herself the 3D printed wristband on his/her left wrist. Since the casing was designed in a flexible soft plastic with a traditional watch bracelet, such a task was reported to be easy achievable by every of our participants. In the mean time, a supervisor, in charge of the data record, initiated a BLE connection between the wearable device and the *Raspberry Pi 3* to pair these two devices together. Next, participants were explained by the supervisor how to achieve properly each exercise of the set described in the previous section. Each daily activity was explained to every participants as well. Then, a short period of time was allowed to participants that required a training on either some exercises or activities to be recognized by our system. This period was obviously not recorded. Once such a practicing phase was completed, the participants were informed which exercise they had to achieve and when to begin performing it through an audio feedback. In these experiments, each exercise as well as each finite daily activity (i.e., *sitting down* and *standing up*) had to be repeated ten times. However, each continuous daily activity such as *walking* and *running* were recorded during 30 s.

## 7. Results

### 7.1. Classification Performance Metrics

Since the performance of the classification task requires to be properly evaluated, it is important to provide representative metrics.

To this end, the accuracy (Acc.) is probably the most dominant measure in the literature because of its simplicity. This measure provides the ratio between the correct number of predictions and the total number of cases given as,

(7) $$Acc.=\frac{TP+TN}{TP+TN+FP+FN}$$

where TP and TN refer to true positive and true negative predictions respectively, and the total additionally include false positive (FP) and false negative (FN) predictions.

Despite its popularity, accuracy alone does typically not provide enough information to evaluate the robustness of prediction outcomes. Indeed, accuracy does not compensate for results that may be expected by luck. Indeed, a high accuracy does not necessarily reflect an indicator of a high classification performance. This is the accuracy paradox. For instance, in a predictive classification setting, predictive models with a given level of accuracy may have greater predictive power than models with higher accuracy. In that sense, as suggested by Ben-David [34], we decided to provide the Cohen’s kappa (*k*) evaluation metric as well. This measure takes into account such a paradox and remains a more relevant metric in classification evaluation such as the method we suggest in this paper. The kappa measure is given by,

(8) $$k=\frac{P_{o}-P_{e}}{1-P_{e}}$$

where $P_{o}$ and $P_{e}$ are the observed and the expected probabilities respectively.

Another well-known metrics which is often used in related literature and which we choose to provide additionally, is the *F-Score* (*F*),

(9) $$F=2\;\cdot \;\frac{Precision\;\cdot \;Recall}{Precision+Recall}$$

Precision and Recall are expressed by (10) and (11) respectively,

(10) $$Precision=\frac{TP}{TP+FP}$$

(11) $$Recall=\frac{TP}{TP+FN}$$

where FP and FN refer to false positive and false negative predictions respectively and TP corresponds to true positive results.

### 7.2. Results Obtained

In real use case conditions, it is easy to imagine that our system including both the wristband as well as the *Raspberry Pi 3*, may be provided by a therapist to his/her patients. In that sense, he or she will have the responsibility to supervise a specific training of the machine learning algorithm in order to ensure the most accurate recognition possible. In that sense, this research have conducted several evaluation of the suggested system to showcase its reliability in terms of recognition accuracy, computation performance as well as power consumption.

The performance for the recognition of exercises and daily activities through inertial data produced by a wristband that embed a 9-axis IMU was evaluated according to several classification analyses. These achievements were achieved in real time by the main computing unit (i.e., *the Raspberry Pi 3*).

**Figure 6:**
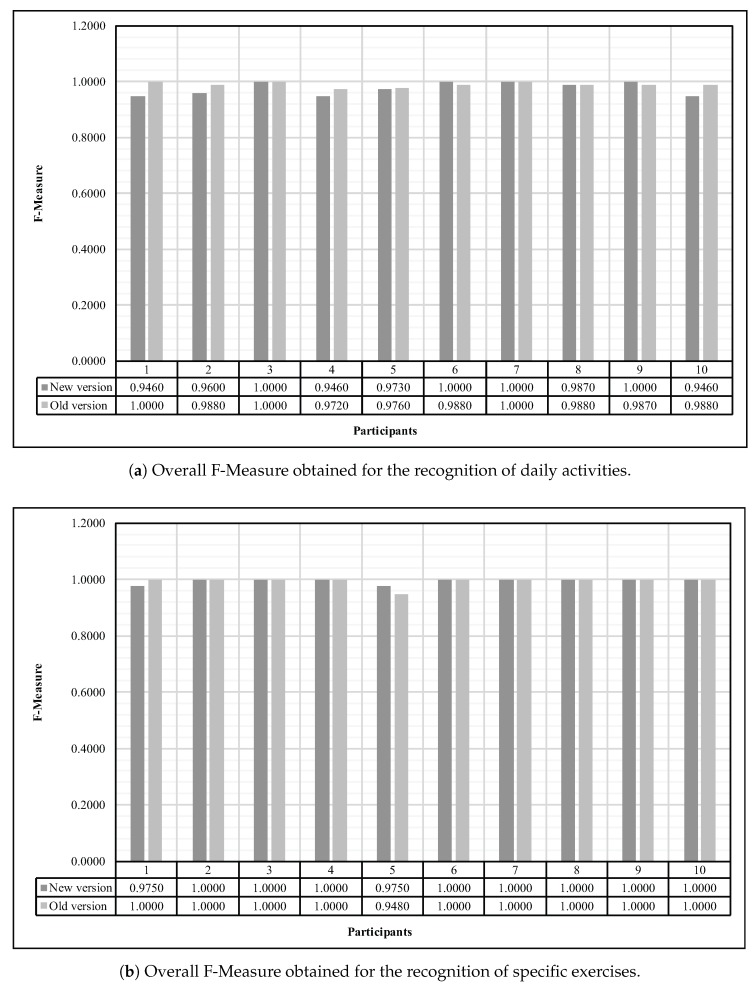
Overall F-Measure obtained for both the recognition of daily activities and specific exercises using the Random Forest algorithm tuned with B=100 trees and $F=\frac{1}{2}\sqrt{m}$ and $F=\sqrt{m}$ features respectively.

First of all, as explained previously, the Random Forest classification algorithm needed to be suitably tuned according to few parameters, The number of trees in the forest *B* and the number of random features *F*. With an empirical approach, we experienced, all the three different parameters ($F=log_{2}\left(m\right)+1$, $F=\frac{1}{2}\sqrt{m}$ or $F=\sqrt{m}$) for the number of random features on models containing from B=50 to B=500 trees where the value of *B* was incremented by 50 between each model construction. Through this evaluation, it occurred that the fittest parameters combination for the recognition of daily activities and specific exercises are $F=\frac{1}{2}\sqrt{m}$ and $F=\sqrt{m}$ features respectively, both with B=100 trees. Consequently, we preserved these parameters to conduct every following assessments. In addition, since this paper suggests a brand new version of the prototype we previously proposed in our previous work [13], all following results that are detailed below also recall the preceding ones. In that sense, Figure 6 exposes the overall F-Measure obtained for both daily activities (Figure 6a) as well as specific exercises recognition (Figure 6b).

However, due to the large amount of features to process, we expressed the necessity to investigate if applying a reduction of their number will either affect the overall recognition rate or not and how to proceed to the reduction of such dimension. To do so, we exploited two different kind of filters. The one that is a supervised method is the *AttributeSelection* algorithm and the second one is the well known Principal Components Algorithm (PCA) that belongs to unsupervised filtering methods. Both algorithms have been tested using Weka software [35] with default parameters. As exposed in Table 2 and Table 3, the remaining number of feature after the reduction process drastically decreased, in both case, and especially with the PCA method. Moreover, it is possible to observe that such a process does not really influenced the overall performance of the recognition.

**Figure 7:**
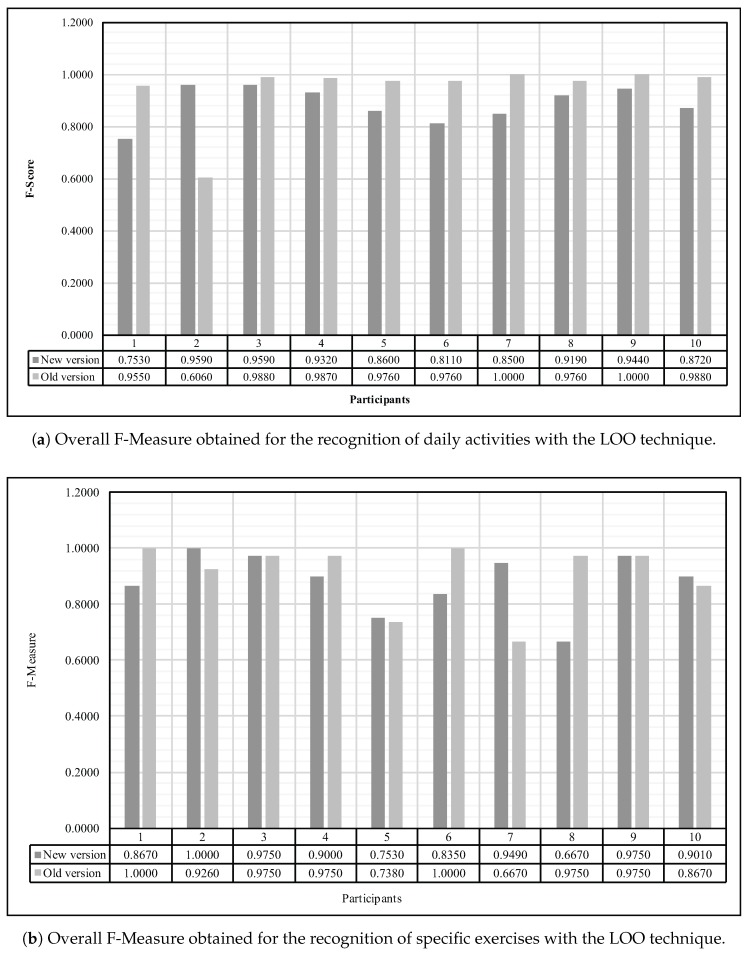
Overall F-Measure obtained for both the recognition of daily activities and specific exercises using the LOO techniques with the same parameter tunning as previously for the Random Forest algorithm.

Presently real use case scenarios only lies in a specific training for each patient supervised by the therapist himself or herself. These situations undeniably led to a high precision in terms of recognition performance but it implies a considerable amount of wasted time for the medical staff. Hence, it appeared valuable for us to experiment the robustness as regards the user variety in order to deliver a system already and identically trained for every patient. To simulate such a situation, a Leave-One-Out (LOO) technique was employed. This process consists on training the machine learning algorithm with the instance of every participants of our experiments excluding a given person and perform the recognition exclusively with instances of this participant left out from the training. As shown in Figure 7 the evaluation of such experiment was made by comparing the F-Measure obtained while performing the LOO technique with our new prototype and the ones obtained with the same method through our previous device for both daily activities (Figure 7a) as well as specific exercises (Figure 7b).

**Figure 8:**
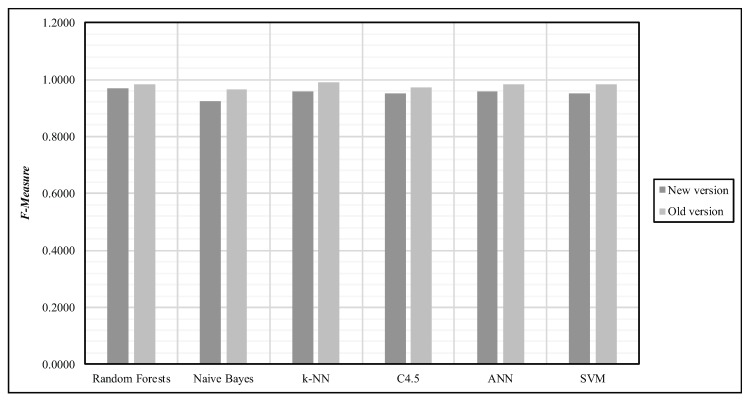
Comparison of our results with the ones that are possible to obtain with others machine learning algorithms where both daily activities and specific exercises are combined.

Although and Random Forest algorithm was selected to classify our data, we have already reported that few other algorithms may achieve such a task accurately. In that sense, we judged relevant to provide a brief comparison of our results with the ones that are possible to obtain with others machine learning algorithms such as *Naïve Baye*, *k-NN*, *C4.5*, *ANN* and *SVM*. In that sense, the Figure 8 provides such a comparison by using the F-Measure as the recognition performance metric where both daily activities and specific exercises are combined.

As a consequence of the two types of activity recognition running on two different window sizes, we investigated how to deal with them. The answer of this problem we found was to run two instances of Random Forest on the same device, each being trained on a different dataset; the first instance was trained with daily activities where the other was dealing the physical exercises. In fact, the two instances are never running at the same time, since the second is only active while the patient is doing its physical training and the first is effective at any time else. The RF recognizing daily activities contains another activity to switch the active algorithm to the recognition of physical exercises. However, while there is an activity to activate the personal training, nothing can stop it on the system. Actually, when the user stops his/her training, the device itself continues to recognize exercises until too much errors occurs (false detections) and then activate back the activity recognition RF. The false detections are managed with a filter based on the energy feature of the physical exercises. In fact, if an exercise is recognized but its energy cannot be related to the average energy recorded in the training dataset for the same exercise, it is considered as a false detection.

### 7.3. Power Consumption Evaluation

The new prototype we suggest in this paper was introduced to cope with the battery life problem we encountered in the first version. Thus, several technologies that are known for their power efficiency were used. In order to demonstrate this improvement, we conduct two energy consumption experiments on both platforms.

The first one consist of recording the life duration when powered by the exact same battery capacity of 400 mAH. While this approach seems naive, it showed some interesting first results. The device based on the *Edison* stayed powered for 2 h and 34 min and the device based on the *nRF52* 22 h and 46 min which is nearly ten times longer.

**Figure 9:**
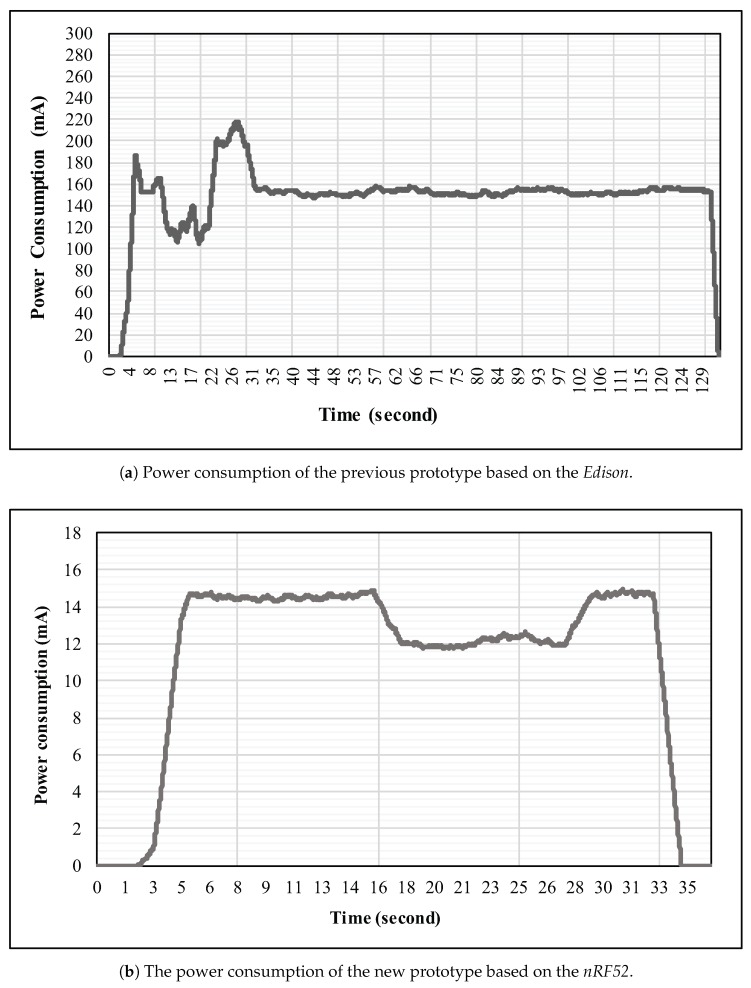
Overall power consumption of both the previous as well as the new prototype expressed in milliamperes.

The second experiment is a more serious one and it involved a full energy analysis of both platforms. To realize such study we used an *INA219* chip from *Adafruit* which allowed us to record the power consumption in milliampere from a stabilized power supply at a fixed sampling rate of 60 Hz. The scenarios used during these experiments were slightly different for each prototype. The Edison started to a shut down state on the first 2 s. Then its complete start up lasted 2 min to allows it to boot from this time, it starts the streaming of the data over WiFi to finally shut down again. On the other hand, the *nRF52* started in off state. Directly after its start up, it begins BLE advertising for a total of 12 s followed by another 12 s of data streaming. Finally it came back to an advertising mode for 8 s before going to an idle mode. The results of these experiments are illustrated in Figure 9 for the *Edison* (Figure 9a) as well as for the *nRF52* platform (Figure 9b), where the ordinate is the power consumption in milliamperes and the abscissa refers to the total duration of the experiment in seconds. The Edison prototype starts with an erratic power consumption going from 75 mA to 275 mA before stabilizing to 150 mA during the streaming phase. In opposition, the *nRF52* shows us two relative flat phases. The first one with an average of 14 mA for the advertising and another one with an average of 12 mA during the data streaming. Similarly to the first experiment, a factor 10 against the Edison between the power consumption of our two platforms was observed.

In order to make our device fully usable by end users, the power consumption issue had to be resolved. By moving from the WiFi to the Bluetooth Low-Energy technology for this new prototype, a power consumption ten times better has been rigorously proven. In that sense, we can now affirm with a certain confidence that our new wristband is ready to move to a commercial version as regards energy consumption.

## 8. Discussion

We have presented three important results in this paper. The first one is the recognition rate observed with the two prototypes. The second one is the results when applying or not the features selection and finally the energy consumption values for the two devices.

Concerning the different recognition rates of our application, they are divided in two main categories depending on the dataset used to do the tests. We notice that neither the leave one out nor the full dataset seems to have noticeable differences in terms of recognition rates depending on the device we used to realize the test. This specific observation tends to prove that our new prototype does not have a major impact on our recognition process even if we drastically decreased the sampling rate (from 100 Hz to 60 Hz) and move to less energy consuming technologies.

The next major result in our comparison is the feature selection process and its impact on the recognition rate. With less than a percent improvement, we clearly demonstrate the minor impact of such process on our datasets. Nevertheless, since this process retains only 20% of the total features it spares nearly 80% of computing power on the Raspberry Pi during the recognition phase which is really interesting in terms of energy consumption and computation power.

Finally, we realized an energy consumption quantification experiment to ensure that our new prototype is really better concerning the battery life. These results reveal that with a ten time longer battery life our new device is clearly better suited for some final tests with end users.

## 9. Conclusions

In this work, we proposed a prototype to replace the previous version [13] we made to recognize both daily activities and physical exercises on the same device. A new experiment has been conducted to confirm the performances of our new prototype. With approximately the same recognition rate than the previous one (98.54% of F-Measure) using Random Forests (RF), we can assert that even with a lower sampling rate, we still achieved excellent results. Furthermore, we significantly improved the battery life of our device. Indeed, while the previous system shut downed after two hours and a half, this new one can be running for approximately a entire day. To verify our power consuming, we realized an energy study published in this work.

In real-life conditions, such a device could be really useful for people suffering from DM1 or any muscle disease, considering it can monitor the realization of the physical program, given by a therapist to a patient, in the comfort of their home. It can also recognize daily activities to quantify them, and notify the therapist how much time the patient spent doing physical activities (walking, running, etc.).

Finally, even if the prototype induces great results, it could be really interesting to collect data with patients affected by muscle diseases. As we wanted this system to be the most automated possible, a huge improvement could be to remove the special activity needed to switch recognition modes from daily activities to physical exercises.

## Figures and Tables

**Table 1:** Description of all exploded part exposed in Figure 1b of our previously proposed system.

Exploded Part	Component	Description
**1b.1**	*Intel^®^ Edison*	*The Intel^®^ Edison* is an ultra small computing platform powered by the *Intel^®^ Atom™* SoC dual-core CPU and including an integrated WiFi and Bluetooth LE connectivity.
**1b.2**	micro SD card block	This block provides additional storage in the form of a micro SD card.
**1b.3**	9-axis IMU block	This block contains an 9-axis IMU (*LSM9DS0*). The nine axes are obtained by combining a 3-axis accelerometer, a 3-axis gyroscope and a 3-axis magnetometer.
**1b.4**	power supply block	This block contains a Lithium Polymer (LiPo) battery of 400 mAh and all the necessary circuitry to manage and charge it.

**Table 2:** Activities.

Algorithm	Remaining Number of Features	Acc.	F1	k
*PCA*	15	0.96	0.95	0.95
*AttributeSelection*	27	0.95	0.95	0.94
**Without feature reduction**	105	0.96	0.96	0.95

**Table 3:** Exercises.

Algorithm	Remaining Number of Features	Acc.	F1	k
*PCA*	14	0.95	0.95	0.93
*AttributeSelection*	19	0.97	0.97	0.96
**Without feature reduction**	105	0.98	0.98	0.97
